# Effectiveness of Virtual Reality–Based Physiotherapy Interventions on Pain, Range of Motion, and Gait Function in a Juvenile Hip Arthritis Patient: A Case Report

**DOI:** 10.1155/carm/5573621

**Published:** 2026-04-30

**Authors:** Satheeskumar Durairaj, Sukumar Shanmugam

**Affiliations:** ^1^ Department of Physiotherapy, College of Health Sciences, Gulf Medical University, Ajman, UAE, gmu.ac.ae

**Keywords:** gait, juvenile arthritis, pain, physical therapy, virtual reality

## Abstract

Gamification has shown promising results in pediatric rehabilitation, but there is a lack of research specifically investigating its effects on hip juvenile idiopathic arthritis (JIA) in children. This case study aimed to explore the impact of gamification‐based rehabilitation combined with conventional physiotherapy on lower limb function and walking activities in a child with hip JIA. An 11‐year‐old female was diagnosed with hip JIA and presented with hip joint pain and difficulty in standing and walking. She underwent a 6‐month intensive conventional physiotherapy program combined with virtual reality (VR)–based gamification using the Walker View treadmill system and D‐Wall digital mirror device. Each treatment session lasted 60 min and was conducted 5 times a week for 6 months. The visual analog scale (VAS), goniometry, Walker View treadmill system score, and Hip Disability and Osteoarthritis Outcome Score (HOOS‐12) outcome measures were used during pretest, posttest, and follow‐up assessments at 0, 6, 9, and 12 months. The combined approach of VR‐based gamification and conventional physiotherapy led to significant improvements in pain, functional range of hip joint movements, and gait in this JIA patient. She also showed significant progress across all outcome measures at different timelines. This case report concludes that combining VR‐based gamification with conventional physiotherapy is effective in improving hip joint function and mobility in children with hip JIA. It is recommended to combine VR‐based gamification with conventional physiotherapy to enhance lower limb function and walking activities in JIA patients, and more future studies are needed to generalize this effect.

## 1. Introduction

Juvenile idiopathic arthritis (JIA) is a chronic inflammatory condition with an unknown etiology that affects children under the age of 16 and persists for at least 6 weeks. It is characterized by persistent joint inflammation, stiffness, and, in some cases, potential systemic manifestations. JIA progression may involve synovitis, articular structure damage, and eventual joint ankylosis. Based on clinical presentation, JIA is broadly categorized into nonsystemic JIA and systemic JIA (1).

Hip joint involvement represents a significant concern in JIA, with reported prevalence ranging from approximately 20%–63% of affected children [1]. The prevalence of hip involvement is similar in both nonsystemic and systemic JIA subtypes [2]. Hip osteoarthritis or avascular necrosis can develop within 2–5 years after the onset of hip JIA, highlighting the need for timely and effective interventions [3].

The standard management for JIA commonly combines pharmacological and physiotherapy interventions. Individualized exercise programs have shown effectiveness in improving muscle strength and physical fitness in children with JIA [4]. Proprioceptive training combined with strengthening exercises registered better functional improvements than strength training alone [5]. Similarly, the previous study supports core stability with strength training, which appears essential for maintaining bone health and maintaining or improving the hip joint function [6]. Although exercise is acknowledged as an important component in improving mobility and functional outcomes, there remains no agreement on the optimal duration, frequency, or exercise intervention for JIA [7]. In addition, adherence challenges that include limited enjoyment, time constraints, and inconsistent parental support often hinder the successful implementation of the conventional physiotherapy interventions [8].

Virtual reality (VR) gamification is a modern technology intervention which provides better interaction in multisensory tasks that are mainly performed in daily life activities, such as motor manipulation, mobility, balance, and higher mental function, with full active involvement of patients [9]. This VR technology provides 3 or 2‐dimensional, computer‐simulated environments to create immersive and nonimmersive experiences for the users. A prior study showed that both immersive and nonimmersive VR therapy produces significant pain reduction in adult musculoskeletal disorders [10]. Another recent study suggests that gamification methods, such as VR and augmented reality, can significantly enhance attention and active participation in pediatric musculoskeletal rehabilitation [11, 12]. A recent systematic review reported reductions in pain and anxiety among juvenile patients, especially for acute‐stage, nonjoint‐specific painful conditions [13]. The hip JIA is a relatively rare and uncommon condition. The hip JIA presents with persistent pain, antalgic gait pattern, and reduced hip loading tolerance, which are clinically more challenging and limit participation in conventional physiotherapy. The VR‐based intervention, such as D‐Wall (DW) and Walker View (WV), provides task‐oriented repetition, movement correction through visual feedback, enhances gradual functional loading, and produces more enjoyable and interactive sessions for JIA patients. Despite the promising results of gamification in pediatric rehabilitation, evidence for VR‐based intervention for hip JIA remains scarce, specifically at the level of complete clinical case details.

Hence, this case report describes the clinical presentation and physiotherapy details of a child with hip JIA who received VR‐based interventions. Therefore, this case report aimed to explore the feasibility, insight into patient‐specific response, and clinical decision‐making associated with gamification‐based VR rehabilitation combined with conventional physiotherapy to improve lower limb function and walking activities in a child with hip JIA.

## 2. Case Presentation

An 11‐year‐old female presented at Thumbay Rehabilitation Hospital, Ajman, UAE, in February 2023, with a chief complaint of bilateral hip joint pain. Informed consent was obtained, and a detailed history was collected from parents. Her present history of illness started with localized pain in both hip joint regions that had started 4 months ago and for which she received analgesic treatment at a local hospital. However, hip joint pain rapidly increased within a week and was aggravated by standing and walking. She was readmitted to a local hospital, and there was no significant medical, family, or psychosocial history. Hematological investigations were unremarkable. Hip MRI demonstrated bilateral avascular necrosis. Based on clinical, laboratory, and imaging findings, and in accordance with International League of Associations for Rheumatology (ILAR) criteria, nonsystemic JIS with hip involvement was diagnosed after exclusion of alternative etiologies. Surgical treatment was recommended, but her parents opted for homeopathic treatment and continued for the next 3 months, which resulted in minimal improvement in hip joint pain and function.

At present, her disease severity level was measured by using the Clinical Juvenile Arthritis Disease Activity Scale (cJADAS), placing her in the moderate risk category with a score of 18/30 [14]. She presented with main complaints of hip joint pain and nonspecific pain over the lateral aspect of the upper to mid‐thigh region on both sides. Hip joint pain intensity was recorded as 7/10 points on the visual analog scale (VAS), which is a reliable tool to assess pain perception [15]. On physical examination, her hip joint range of motion, measured by using goniometric measurement [16], showed limited active and passive hip joint range of motion in all directions with painful end feel in both hip joints. Manual muscle testing demonstrated bilateral hip joint muscle weakness (Grades 2/5). She was independent in sit‐to‐stand, can stand without support for 10 min, and walks with support. Detailed gait analysis was performed by using the “WV” treadmill system [17], which found altered gait parameters such as step length, foot contact time, center of gravity (CoG) control, load symmetry, and walking speed, with reduced trunk and hip range of motion. In addition, the Hip Disability and Osteoarthritis Outcome Score‐12 (HOOS‐12) was used to measure functional limitation. HOOS‐12 was selected because the primary aim of the case report was to describe the hip‐specific functional impairment and quality of life (QOL) impact, rather than to assess overall JIA disease activity. The HOOS‐12 contains 3 domains: pain, activities of daily living (ADL), and QOL, with 4 question items in each domain. Each question’s score ranges from 0 to 4, where 0 denotes no hip problems and 4 denotes extreme hip problems. Each domain’s total score is converted to a scale of 0–100 to quantify person‐specific hip joint problems. A minimally clinically important change score of more than 9 points indicates the usefulness of the intervention in the HOOS‐12 scale [18, 19]. The patient’s initial HOOS‐12 scores were 44 for the pain domain, 63 for the ADL domain, and 38 for the QOL domain. These baseline assessment details are given in Table 1. Subsequently, she began intensive physiotherapy to improve her hip joint range of motion and gait function.

**TABLE 1:** Outcome score of pain, range of motion, gait parameters, and functional activity on pre, post and follow‐up timelines.

Variables				Pretest	Posttest	Follow‐up	
				Day 1	6th month	9th month	12th month
Pain intensity: VAS (1‐10 points)				7	1	0	0

Hip joint passive range of motion (goniometer)		Flexion (degrees)	Left side	30	75	80	90
			Right side	30	80	80	100
		Abduction (degrees)	Left side	10	20	30	40
			Right side	10	30	30	40

^∗^Gait parameters	Kinematic	Trunk movements range (degrees)	Flexion–extension	4.8	2.9	3.6	3.1
			Lateral flexion (L‐R)	10.8	8	7.9	5.3
		Hip joint’s flexion‐extension range (degrees)	Left side	22.9	25.6	37.6	51.8
			Right side	20.9	35.7	43.4	55.3
		Knee joint’s flexion‐extension range (degrees)	Left side	33.2	37.4	52.4	42.6
			Right side	21.7	40.7	51.3	50.4
		Step length (centimeters)	Left side	39	62	65	65
			Right side	23	41	63	67
	Kinetic	Limb contact time (seconds)	Left side	1.99	0.57	0.43	0.57
			Right side	2.13	0.64	0.45	0.6
		Load symmetry (%)		6.6	1.3	2.7	3.9
		Average gait speed (kph)		1	3.5	4.7	4.9

Activities of daily living: HOOS‐12 (0–100)			Pain	44	12	8	6
			ADL	63	45	35	30
			QOL	38	20	18	18
			Overall	48.3	25.7	20.3	18.0

User Experience Questionnaire				—	98	—	—

*Note:* HOOS‐12, Hip disability and Osteoarthritis Outcome Score.

Abbreviations: kph, kilometer per hour; L‐R, left to right; RoM, range of motion; VAS, visual analog scale.

^∗^Obtained using the Walker View treadmill system.

### 2.1. Interventions

**FIGURE 1:**
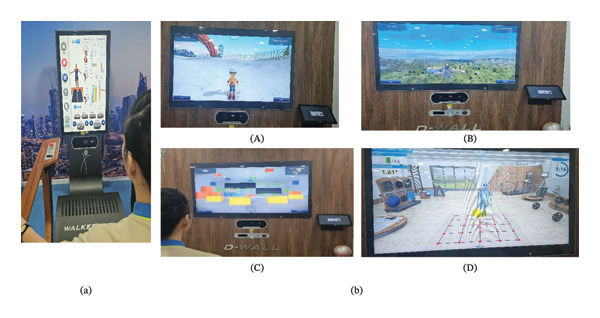
Virtual reality–based interactive rehabilitation modules. (a) Walker View system displaying real‐time gait analysis, including temporal–spatial parameters, body alignment, foot pressure distribution, and an on‐screen avatar mirroring the user’s movements for gait retraining. (b) D‐Wall interactive virtual‐training modules demonstrating various functional tasks: (A) ice skating module for balance and lower limb coordination; (B) flight driving module targeting postural control and upper‐body movement accuracy, (C) target shooting module promoting hand–eye coordination and dynamic balance, and (D) agility training module providing multidirectional movement drills with motion‐capture feedback.

The patient’s treatment protocol started with 20 min of conventional physiotherapy, which included 5 min of passive movement to hip joints, 5 min of stretching for hip flexors and abductors, and 10 min of strength training for hip abductors and extensors. The resistance load was determined using 1‐repetition maximum (RM), with the patient performing 8–10 repetitions per set for 3 sets, taking 20 s of rest between each set. The resistance load progressively increased every 4 weeks [20]. Later, she underwent two VR‐based interventions (Figure 1): the first with the WV equipment (TecnoBody SRL, Italy) for 10 min. WV is a VR‐based treadmill system that provides immediate feedback on balance and locomotion. Following this, she underwent the DW equipment (TecnoBody SRL, Italy) [21] for 20 min. DW is a digital mirror device that provides immediate feedback through VR‐based mirror therapy and game‐based exercises. Nonimmersive VR‐based games (ice skating, flight driving, target shooting, and agility training) in DW were selected for lower limb rehabilitation, and all the games were selected based on the machine dynamic activity (MDA) framework [22].

A comprehensive home exercise program was designed to maintain hip joint flexibility, and the patient’s adverse reactions and adherence to the treatment protocol were monitored using a provided daily log. The training started at low speed with adequate rest periods, and progress was made by increasing speed and reducing rest periods over the next 6 months.

**FIGURE 2:**
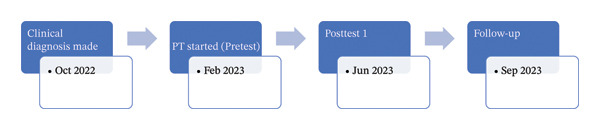
Patient condition timeline details in schematic representation.

The VAS, goniometer, VR‐based gait function in WV, and HOOS were reassessed at the 6th month, and the follow‐up assessments were conducted in the 9th month and at the end of the 1st year. In addition, the User Experience Questionnaire (UEQ) [23] was used to record the satisfaction level of the patient on the VR‐based gamification at the 6th month postintervention. The UEQ has 8 items, and each item is rated on a 0–6 point scale. The total score is 48, which is converted into a percentage to determine the patient’s level of satisfaction. According to the postintervention outcome data, as shown in Table 1, significant improvements were observed across all the outcome measures at the end of the 6th month. Also, the improvements were either maintained or improved further at the follow‐up period at the end of the 9th month and the end of the 12th month. These observations suggest that VR‐based intervention combined with conventional physiotherapy is useful to reduce pain, improve joint mobility, encourage better gait function, and increase the QOL in hip JIA for children. The complete patient details are represented in a pictorial timeline in Figure 2.

## 3. Discussion

This case report presents a novel approach to treating JIA by combining VR interventions with conventional physiotherapy and home‐based programs. The studies have focused on improving the functional range of hip joints in JIA patients by using various conventional physiotherapy interventions and advanced technology efficacy and raising concerns over the need for a comprehensive intervention protocol [20, 24]. So, this case report addressed this gap and attempted to register the effects of advanced technology on hip joints in JIA patients.

This patient showed good adherence to the PT session appointments and registered a satisfaction level of 98% in the UEQ scale for VR‐based training sessions, which could be the answer for the commonly registered low motivation for conventional exercise programs. Also, she did not register any adverse reaction, discomfort, or any pain exacerbation for the VR‐based interventions [25].

The combination of VR and conventional physiotherapy produced a trend toward improvement in hip joint pain and functional joint range of motion, as reflected in the VAS, goniometer, VR‐based gait parameters, and HOOS‐12 scores. During the initial phase of treatment for up to two months, passive mobilization and conservative stretching exercise played a crucial role in reducing pain and restoring the initial joint range of motion. As therapy progressed, active‐assisted exercises, strength training, and VR‐based activities helped to maintain these gains and further improvements in the hip joint range of motion and gait performance. Similar to our case report, previous studies have registered the remarkable evidence that well‐structured or targeted conventional physiotherapy is effective in pain control, increasing joint mobility, enhancing daily life activities, and improving aerobic conditioning [24]. Especially, a systematic review that concluded physical therapy interventions effectively improve self‐rated pain and joint range of motion in hip joint disorders. Specifically, this systematic review highlighted that joint mobilization for pain reduction and strengthening exercises for improving muscle balance are primary factors in increasing hip joint function [26].

Throughout the intervention period, the patient received multiple therapeutic components concurrently, including passive mobilization, stretching, active‐assisted exercises, strengthening, and VR‐based activities. Consequently, it is not possible to attribute the observed changes to any single intervention component, including VR‐based activities. These findings should therefore be interpreted as descriptive and exploratory, highlighting the potential feasibility of a combined intervention approach rather than demonstrating the independent or causal effect of VR.

This case report also demonstrated improvements beyond hip joint function, extending to gait function and ADL. The HOOS‐12 score improved beyond its minimally clinically important change score, indicating clinically significant progress for VR‐based conventional physiotherapy interventions. These results are consistent with previous studies that have shown the effectiveness of VR‐based interventions in reducing pain and improving QOL in various musculoskeletal conditions [22]. Although HOOS‐12 was originally developed for adult hip pathology, it was selected for its hip‐specific domains. However, its use in pediatric populations should be interpreted cautiously.

VR‐based intervention in this case has contributed to significant findings through a few mechanisms. Real‐time feedback during VR therapy could have produced immediate movement correction that potentially enhanced motor control of extremities and reduced compensatory gait patterns commonly observed in hip involvement. The immersive, participative, and dynamic nature of VR may also have improved motivation and involvement, which are the most essential factors in long‐term rehabilitation, particularly in younger children. Also, the ability to perform repeated task‐specific training in a controlled virtual environment may have helped the patient to achieve functional mobility. These elements have been identified in previous research as key components of effective VR‐based rehabilitation programs [27, 28]. Altogether, the improvements in these outcomes suggest that the integration of VR‐based intervention with conventional physiotherapy could provide not only physical improvements but also advantages in psychological and motivational aspects to increased adherence and overall treatment effectiveness.

The coexistence of hip involvement attributed to JIS and radiological features of avascular necrosis introduces diagnostic uncertainty. Nevertheless, the observed clinical trends highlight the potential importance of physiotherapy in supporting pain management, mobility, and functional adaptation, although the relative contributions of inflammatory control, mechanical recovery, and rehabilitation cannot be distinguished in this single case.

Even though this case report presents promising results for the use of VR in orthopedic rehabilitation, particularly for lower limb function in JIA patients, it is important to acknowledge the limitations such as it being a single case report without a control group; it cannot provide comprehensive evidence of VR’s effectiveness. Future studies with larger sample sizes and control groups are necessary to fully evaluate the impact of VR interventions across various musculoskeletal disorders.

In conclusion, combining VR‐based gamification with conventional physiotherapy is beneficial in improving hip joint pain, function, and mobility in children with hip joint JIA. This case report offers preliminary observations on the combined use of VR and conventional physiotherapy in JIA and highlights the need for further research to clarify its potential role in pediatric rheumatology and orthopedic rehabilitation.

## Author Contributions

Satheeskumar Durairaj: preparation, creation, and/or presentation of the published work, specifically writing the initial draft (including substantive translation).

Sukumar Shanmugam: preparation, creation, and/or presentation of the published work by those from the original research group, specifically critical review, commentary, or revision, including pre‐ or postpublication stages.

## Funding

No funding was obtained for this study.

## Consent

Informed written consent was obtained from the patient’s parents for the publication of this case report.

## Conflicts of Interest

The authors declare no conflicts of interest.

## Data Availability

All data generated in this case report are included in this article. Additional information is available from the corresponding author upon reasonable request, in accordance with patient privacy and institutional policies.
